# Perceptions of sulphadoxine-pyrimethamine use among pregnant women in sub-Saharan Africa: a scoping review

**DOI:** 10.5281/zenodo.7828460

**Published:** 2023-04-06

**Authors:** Patricia Ogba, Oluwaseun Badru, Bonny Ibhawoh, Norm Archer, Andrea Baumann

**Affiliations:** 1 Faculty of Health Sciences, Global Health Office, McMaster University, Main St. W, Hamilton, Ontario, Canada; 2 Usmanu Danfodiyo University Teaching Hospital, Sokoto, Nigeria; 3 Department of History, McMaster University, Main St. W, Hamilton, Ontario, Canada; 4 Degroote School of Business, McMaster University, Main St. W, Hamilton, Ontario, Canada.

## Abstract

**Background:**

Malaria is a major global public health issue that disproportionately affects pregnant women in sub-Saharan Africa. The World Health Organization recommends intermittent preventive treatment with sulfadoxine-pyrimethamine (IPTp-SP) for its control. Despite its proven efficacy, drug uptake remains low. Sulphadoxine-pyrimethamine (SP) safety concerns have been cited as one of several reasons for this low uptake.

**Methods:**

We conducted a scoping review using the Arksey and O'Malley framework and the health belief model to investigate perceptions of SP use among pregnant women in sub-Saharan Africa. We looked for peer-reviewed publications in five international databases.

**Results:**

The review included 19 articles out of a total of 246. It showed that pregnant women in sub-Saharan Africa have a good understanding of malaria and its consequences, but this does not necessarily translate into increased IPTp-SP uptake. It is worrisome to know that some pregnant women (from 2 studies) did not believe that SP use is beneficial, and several participants (from 4 studies) were unsure or did not see the drug as an effective intervention. Many pregnant women believe SP harms them, their partners, or their unborn children.

**Conclusions:**

Healthcare professionals should continue prescribing and encouraging pregnant women to use SP for malaria prevention until a better substitute becomes available.

## Introduction

Malaria is a significant global health problem that disproportionately affects children and pregnant women [1,2]. In 2021, there were about 247 million malaria cases worldwide. Although malaria deaths decreased steadily from 897,000 in 2000 to 568,000 in 2019, they increased to 619,000 in 2021. This increase in deaths was attributed to the COVID-19 pandemic's disruption of essential malaria services [3]. The African Region of WHO bears a disproportionately large share of the global malaria burden. Between 2019 to 2021, this region experienced an increase from approximately 94% of all malaria cases and deaths to 95% of cases and 96% of deaths worldwide [2-6]. Due to the high endemicity of malaria and repeated exposure to this infection in sub-Saharan Africa (SSA), people tend to develop immunity to it during the early years of their lives. Despite this immunity, pregnant women are highly susceptible to malaria, especially in their first and second pregnancies, because of lowered immunity associated with pregnancy [1,7,8].

Malaria is the third leading cause of death among women of reproductive age in SSA [2,4]. In 2021, there was an estimated 40 million pregnancies in 38 SSA countries, with 13,3 million (32%) exposed to malaria infection, causing devastating maternal and neonatal outcomes [3]. Annually, among other consequences, malaria in pregnancy (MiP) is responsible for 2-15% of cases of severe maternal anaemia, 8-14% of low birth weight (LBW), and 20% of stillbirths. Also, 11% of neonatal deaths, 10,000 maternal mortality, and 100-250 thousand foetal deaths are attributed to MiP [1,2,5,7]. Recognising the adverse consequences of MiP, WHO recommends a three-pronged approach to its control. These include malaria case management, the use of insecticide-treated nets (LLINs), and IPTp-SP [4,9,10]. IPTp-SP is administered in at least three SP doses to all pregnant women during each scheduled antenatal care (ANC) visit, regardless of whether they have malaria. It is administered monthly, starting from the second trimester, after quickening, until delivery [4,10-12]. IPTp-SP is currently rated as a beneficial and cost-effective measure for controlling MiP and its adverse impacts on pregnancies. This intervention has been shown to reduce the incidence of preterm birth, LBW, neonatal mortality, maternal anaemia, and placental parasitaemia when used optimally [5,9,13,14]. During the Roll Back Malaria (RBM)^1^ summit in Nigeria in 2000, African Heads of State pledged that by 2005, at least 60% of pregnant women in malaria-endemic areas should have access to IPTp-SP. This target was increased to 80% by 2010 [4,15-17].

Despite the proven efficacy of IPTp-SP and its adoption as a national policy in 35 SSA countries by the end of 2008, its uptake remains low, falling short of the projected 80% coverage by WHO [4,12,15-18]. In 2013, an estimated 15 million of SSA's 30 million pregnant women did not receive a single dose of IPTp-SP [18]. The overall percentage of pregnant women who received three doses of IPTp-SP ranged from 13 to 19% between 2014 and 2016, while in 2018 more than 60% did not receive the recommended IPTp-SP doses [4,15-17]. Among several factors, SP safety concerns have been cited as a cause for this low uptake [1,19-24].

This review builds on the Ogba *et al.* [12] review that showed that pregnant women's perception of preventive malaria interventions is a significant determinant of their uptake. This finding necessitates further investigation of pregnant women's perception of SP uptake. To the best of our knowledge, there are only scant data on the holistic evaluation of the perceptions of SP use among pregnant women in SSA. Using the Arksey and O'Malley framework [25] and some elements of the health belief model (HBM) [26], this scoping review therefore examined the perception of malaria severity and SP use among pregnant women across SSA. The HBM is a psychological and behavioural framework for investigating health behaviours. This framework has been successfully used in health promotion and disease prevention programmes [7,27-29] and helps to understand the failure of people to accept intervention strategies [26]. This model suggests that a person's perception of the efficacy and benefits of a recommended health behaviour are essential predictors of such behaviour adoption [7,26]. This model is suitable for examining the perceptions of SP use for MiP control among pregnant women. We anticipate that the study findings will highlight the importance of healthcare professionals continuing to educate the general public and pregnant women about current malaria prevention approaches and dispel their misconceptions about IPTp-SP.

## Materials and Methods

This scoping review followed a five-stage methodological framework that included (1) identifying the research question and (2) relevant studies, (3) selecting the studies according to inclusion criteria, (4) charting and interpreting data, and (5) summarising and reporting of results [25]. It was complemented with the Preferred Reporting Items for Systematic Reviews and Meta-Analyses extension for Scoping Reviews (PRISMA-ScR) checklist [30]. We developed the checklist to increase methodological transparency and guide the reporting of this review. We created a scoping review protocol to conduct the study process. This protocol is available upon request. Our research question was broad, i.e., “What is the perception of SP use among pregnant women in sub-Saharan Africa?”

### Search strategy

We conducted a comprehensive literature search to identify all relevant studies reporting on the perception of SP use among pregnant women in SSA. We searched five international databases for peer-reviewed publications, including Ovid Embase, global health, Ovid Medline, Web of Science, and Malaria in pregnancy library. The search took place between December 2021 and January 2022, with the last search done on January 11, 2022.

The search strategy included a range of relevant combinations of keywords: ("perception" OR "attitude" OR "awareness" OR "viewpoint" OR "opinion") AND ("sulphadoxine-pyrimethamine" OR "intermittent preventive treatment" OR "intermittent preventive therapy" OR "IPTp-SP" OR "Fansidar") AND ("malaria in pregnancy" OR "pregnant women" OR "parturients" OR "gravid women") AND ("sub-Saharan Africa" OR "Africa south of Sahara”). Articles were included if they discussed the perceived benefits, efficacy, and risks of SP use among pregnant women in SSA. Books or book chapters and articles that addressed the use of other antimalarials, focusing on children and infants or pregnant women who are HIV positive and have sickle cell anaemia, were excluded. Additionally, articles were excluded for non-availability of full text and if another source better covered the same information. Only articles in the English language were included. To reduce the potential for reviewer bias, study selections were conducted in two stages. First, using predefined inclusion and exclusion criteria, the first author (PO) and co-author (OB) independently screened the titles/abstracts and full text through Covidence, an online reviewing platform [31]. Following that, we resolved differences in study selection through consensus. The study selection process was reported using a Preferred Reporting Items for Systematic Reviews and Meta-Analysis (PRISMA) flow chart [32].

### Charting the data

Two reviewers (PO and OB) extracted data from each publication using an explicitly structured data sheet developed by the authors (see Table 1). Included articles were categorised based on the information they provided. We pulled the following information from each included study: (1) author(s), (2) title, (3) study objective, (4) publication year, (5) geographical location, (6) study design, and (7) key findings.

**Table 1.** Data extraction template.

S/N	Variable	Description
1	Authors	Names of authors that published paper
2	Year of publication	Year in which paper was published
3	Title	Title of paper
4	Aims/objectives	The aims/objectives that guided the conduct of the study
5	Study design	Type of study design
6	Study population	Participants of the study
7	Key findings	Information from the study relevant to the review question/objective

## Results

**Figure 1.**
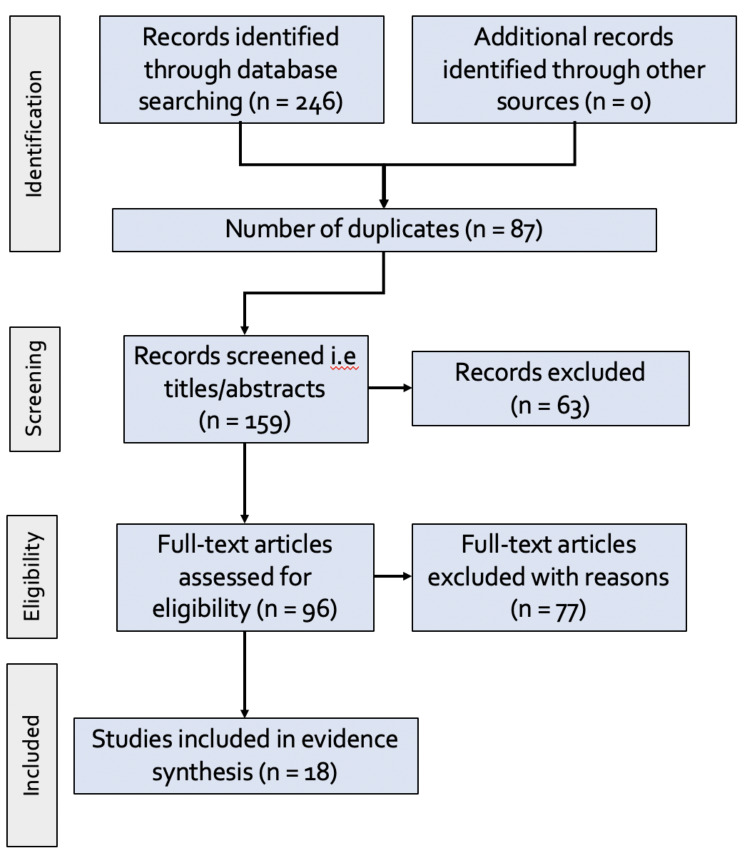
PRISMA flow chart for analysed studies.

A total of 246 references were exported from consulted databases into Covidence for removing duplicates and study screening. We identified 159 articles after removing duplicates. After screening the title and abstract, we selected 96 documents for further analysis by reading the full text. Finally, 18 studies met the inclusion criteria and were selected for data extraction (Figure 1). The included articles represented studies from 9 SSA countries: Congo (n=1), Ghana (n=4), Kenya (n=2), Malawi (n=1), Mali (n=1), Mozambique (n=2), Nigeria (n=8), Tanzania (n=1), and Uganda (n=3). Multiple article types, such as cross-sectional surveys, were present in the included titles.

**Figure 2.**
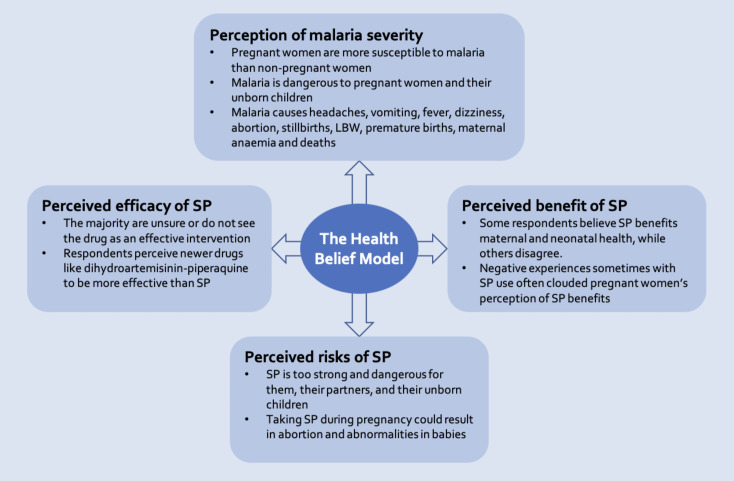
An adapted health belief model (HBM) showing pregnant women’s perception of SP use.

When categorised into the HBM elements adapted for this review, findings from the included papers were classified under "perception of malaria severity", "perceived benefits of SP", “perceived efficacy of SP", and "perceived risks of SP" (Figure 2), which are outlined below.

### Perceptions of malaria severity

We mapped findings from 9 studies to "perceptions of malaria severity". These studies highlighted how pregnant women perceived MiP. According to most studies reviewed [4,18,32-38], respondents were aware that mosquito bites could predispose them to malaria and that pregnant women were more susceptible to malaria than non-pregnant women. Generally, except for some respondents in one Mozambican study [36], MiP was considered dangerous and a serious threat to pregnant women and their unborn children [33,34,35,38]. In one study conducted in south-west Nigeria, some respondents perceived MiP as typical during pregnancy with adverse outcomes. Others attending Traditional Birth Attendant (TBA) clinics believed that pregnant women do not get malaria [4].

Regarding MiP consequences, one study conducted in Nigeria [35] reported poor knowledge of the impacts of MiP among its respondents. In other studies, respondents identified headaches, vomiting, fever, dizziness, abortion, stillbirths, and LBW as MiP impacts [33,34,35,37]. Pregnant women in other studies identified maternal anaemia and deaths, premature births, and neonatal jaundice as the outcomes of MiP [4,18,35,36,38]. Recognising the harmful effects of MiP on the mother and the child, women agreed that it was important to avoid malaria during pregnancy [38]. However, in a study conducted in Uganda, some women did not consider MiP prevention a critical issue [41].

### Perceived benefits of SP

Seven studies included in this review discussed pregnant women's perception of SP benefits. The respondents in five studies [4,34,41-43] believed that SP protects them and their unborn babies from malaria and its adverse effects. Some benefits of using SP recognised by pregnant women included improved health, low rates of maternal morbidity and stillbirths, the delivery of healthy babies, improved baby birth weights, and reduced maternal anaemia [42]. According to the participants, these positive experiences have motivated continued SP use [41,42]. The participants in one study [34] perceived SP to be beneficial to them and their unborn children; however, their negative experiences with SP use often clouded their perception of SP benefits.

One study conducted in Nigeria [33] showed that women who attended ANCs did not use the SP prescribed to them. They claimed they did not need to use the drug since they were not sick. Another study in Nigeria found differences in pregnant women's perceptions of SP benefits. While some respondents believed that SP was perfect for their pregnancies, others had reservations about its use [44].

### Perceived efficacy of SP

Findings from six studies were linked to the perceived efficacy of SP, which discusses pregnant women's perspectives on the effectiveness of SP in malaria prevention. Only 2 studies [34,38] found that pregnant women believe IPTp-SP is effective at preventing malaria. According to these respondents, SP protects the mother and child from MiP and its negative consequences, notably miscarriage and LBW [34,39]. They credited SP use for MiP prevention with their ability to carry their pregnancies to term and give birth to healthy children [34]. Few participants in two studies [33,35] opined that SP use as one of the malaria preventive measures during pregnancy was no longer potent. Two studies [35,37], one in Uganda and one in Kenya and Mali, reported contrasting views of pregnant women on SP effectiveness. While some pregnant women in Uganda agreed that SP was an adequate antimalarial and were willing to retake it, others found it ineffective, particularly for lowering malaria fever, and chose other drugs like dihydroartemisinin-piperaquine (DP) [37,46]. In Kenya and Mali, some pregnant women expressed concern that SP was no longer effective in treating malaria. Other women in Mali had mixed feelings about the effectiveness of IPTp-SP, with some claiming that it was both effective and ineffective at preventing MiP. Respondents in both areas thought that newer drugs, such as Coartem, were more effective than older drugs, such as SP [35].

### Perceived risks of SP

All the papers included in the study reported on pregnant women's perceptions of SP risks and safety. Many pregnant women believe SP is too strong and dangerous for them, their partners, or their unborn children [6,35,41,44,46,47]. While some women got this perception from their previous experiences with SP use [34,36,38], others claimed to have heard their friends or relatives talk about the unpleasantness associated with SP use [38,42]. Women's experiences with side effects varied according to IPTp-SP dose. These women did not necessarily experience side effects with each dose; some did with the first SP dose and others with subsequent use [34,41,42]. Only a few respondents from two studies felt that SP was safe because they had no negative experiences with its use [37,38].

Dizziness, nausea, vomiting, weakness, fever, skin rash, loss of appetite, itching, palpitations, frequent urination, and stomach pain were among the most common side effects mentioned by study participants, according to several articles [4,18,33,34,36, 41-43]. Some women believed that taking SP during pregnancy could result in abortion and abnormalities in babies [41,46]. Some pregnant women were unsure whether their reactions were caused by SP alone or by the presence of other ailments. The majority also believed they reacted to the drug because they took it without eating [35,38,39,40,41]. While these side effects deterred some women from taking or retaking SP during pregnancy [6,35,37, 41], others did not, particularly when it was prescribed by health care professionals (HCPs). The latter believed that receiving drugs from trained HCPs in a hospital made SP trustworthy [34,36,37,40,41,43,44,48].

## Discussion

From this review, it is evident that even when pregnant women perceive MiP as a severe risk or illness, their perceptions toward specific interventions may influence the uptake of preventive measures. The perceived benefits, efficacy, strength, and side effects of IPTp-SP impact women's willingness to use it during pregnancy, as suggested by the HBM.

This review showed that pregnant women in SSA have good knowledge of malaria and its adverse consequences on them and their pregnancies. This sound knowledge, however, does not necessarily translate into improved IPTp-SP uptake [4,12,15-18]. This outcome therefore suggests that low SP uptake is not due to a knowledge deficit. Pregnant women may need further education on the benefits of preventing MiP. HCPs could use educational materials such as pamphlets, pictures, or figures to inform pregnant women about the benefits of protecting themselves and their unborn babies against malaria. As documented in a Ugandan study [49], increased awareness of the benefits of preventing MiP could lead to higher acceptability and use of IPTp-SP.

Numerous studies [5,9,13,14,42] have shown that IPTp-SP benefits pregnant women and their unborn babies. IPTp-SP is associated with improved birthweight, lower maternal anaemia, and lower malaria-related morbidity and mortality rates [5,9,42,50]. Despite these benefits being jeopardised by rising SP resistance across SSA [5,45], it remains the drug of choice for preventing MiP [35,50]. According to this review, most pregnant women believe that using SP during pregnancy is beneficial, which is encouraging. However, reports of a few pregnant women who do not think SP use is helpful are worrisome. These women are unlikely to use IPTp-SP, thus exposing themselves and their unborn babies to malaria and its adverse consequences. This finding emphasises the importance of educating community members, particularly pregnant women, about the benefits of SP during pregnancy. HCPs could even encourage women who had used SP and benefited from it to mobilise other women. A study conducted in Uganda [41] confirms this approach.

This review shows contrasting opinions of pregnant women on the efficacy of SP. The majority are either unsure of its effectiveness [35,37] or do not perceive the drug as an effective intervention [33,35]. Only a few pregnant women believe SP to be efficacious in preventing MiP [34,38]. This belief may be related to HCPs' tendency to prescribe other antimalarials to pregnant women because they do not believe SP is effective, as previously reported [12,20,33,51-54]. This perception and practice among HCPs could also be due to clinical trials showing increasing resistance of malaria parasites to SP throughout SSA [45,55].

Early clinical trials of SP use during pregnancy revealed a significant reduction in placental malaria and its consequences [45]. Nonetheless, recent research has found that IPTp-SP effectiveness has decreased in areas where highly resistant parasites are prevalent [45,55]. Although other candidate therapies, such as dihydroartemisinin-piperaquine, are still being investigated, and despite waning SP efficacy due to increasing parasite resistance, SP remains an essential component of MiP prevention in malaria-endemic areas [10,35,45,50,56]. This review highlights the need for HCPs to continue prescribing and encouraging pregnant women's use of SP for MiP prevention until there is a better replacement, irrespective of their personal beliefs.

Complaints from pregnant women about unpleasant side effects or that the drug is 'too strong' tends to affect compliance, suggesting that women are not adequately informed about SP during their ANC visits. While mild and brief side effects, including nausea, vomiting, and dizziness, may occur when first using SP, this medication is generally well tolerated [10,12]. To improve pregnant women's understanding of SP safety and to dispel the myth that SP, while effective, is too strong to be safe, they should receive information on the safety and side effects of pregnancy drugs at the ANCs [35]. This education has to go beyond the clinics to the communities, especially in rural areas, to reach all community members and the women who do not visit hospitals during pregnancy.

This review shows that while many pregnant women avoid taking or retaking SP due to its side effects [4,6,35,37,41], some women still complete the drug dosage prescribed. These women's continued use of SP demonstrates their trust in HCPs [34,36,37,40,43,44,48]. Previous studies [4,9,12,35] have discussed how the hostility of HCPs, among several factors, has been a barrier to SP acceptance and utilisation among pregnant women. This finding accentuates the vital role of HCPs in increasing SP uptake among pregnant women. For clients to trust and follow their advice, HCPs must provide high-quality antenatal services in a welcoming environment. These findings emphasise the importance of training and retraining HCPs in delivering excellent client care services.

### Limitations

While our study is the first to present a comprehensive overview of pregnant women's perceptions of SP use in SSA, it has some limitations. We made sure that the review included all relevant literature. However, we may have missed some articles because some journals are not indexed in related databases. Despite the framework's recommendation, we excluded grey literature and consultation with key stakeholders due to financial and time constraints. Two reviewers identified and extracted data from relevant studies using pre-determined inclusion and exclusion criteria to reduce error. Although we conducted a thorough search during this scoping review, our inclusion criteria restricting to articles published in English represents a limitation because it may result in language bias. Nevertheless, the variety of journals included contributes to the review's strength.

## Conclusion

Despite the proven benefits of IPTp-SP, uptake in SSA remains low, falling short of WHO's target of 80% coverage. Governments in SSA must continue to prioritise increasing SP uptake among pregnant women. There should be ongoing education to help pregnant women to understand the risks of MiP to themselves and their unborn babies and to dispel myths about using IPTp-SP. HCPs should prescribe SP and emphasise that research shows that the benefits offset its side effects. Providing mothers with more education emphasising the safety and benefits of SP would encourage them to view this therapy as effective, consequently increasing its uptake. Finally, more research into other useful MiP prevention drugs should be conducted to ensure suitable drug replacements for SP if its efficacy falls below what is currently available and acceptable.

## Footnotes

1 The World Health Organization, the World Bank, UNICEF, and UNDP launched the Roll Back Malaria (RBM) initiative in 1998. This pledge, which aims to cut malaria deaths in half by 2010, was endorsed by African heads of state in 2000 at a summit in Abuja, Nigeria [57].

## Appendix A

To generate search terms, the research question was broken down into keywords. An extensive list of synonyms and related words was identified from these keywords, using as many terms as possible. Synonyms and related terms for each category were combined using Boolean operators "OR" and "AND".

In complying with the PRISMA-ScR checklist, the search query for the database OVID is provided below: (“Perception” OR “attitude” OR “awareness” OR “viewpoint” OR “opinion”) AND (“Sulphadoxine-pyrimethamine” OR “intermittent preventive treatment” OR “intermittent preventive therapy” OR “IPTp-SP” OR “fansidar”) AND (“Malaria in pregnancy” OR “pregnant women” OR “parturients” OR “gravid women”) AND (“sub-Saharan Africa” OR “Africa south of Sahara”).

## Appendix B

**Table:** The characteristics of the studies included in this review are listed Included literature after a systematic search

First author (Year)	Reference number	Title	Aims/Objectives	Geographical location	Study design	Key findings
Adeniran *et al.* (2018)	18	Intermittent preventive therapy in pregnancy with sulfadoxine/pyrimethamine for malaria prophylaxis among parturients in Ilorin, Nigeria.	To assess the knowledge, attitude, and factors associated with the use of IPTp-SP among antenatal clinic attendees in Ilorin.	Nigeria	Quantitative	Many clients believed malaria was dangerous during pregnancy, causing maternal anaemia, miscarriages, and intrauterine foetal death. Respondents also expressed dissatisfaction with how SP made them feel weak, dizzy, and nauseous. Some of them vomited after using SP, while others did not.
Akinleye *et al.* (2009)	47	Knowledge and utilization of intermittent preventive treatment for malaria among pregnant women attending antenatal clinics in primary health care centers in rural southwest, Nigeria: a cross- sectional study.	To assess the use of IPTp among pregnant women attending primary health centres in the rural area and determine factors that influence the uptake.	Nigeria	Quantitative	Respondents in this study expressed concern that the drug used for IPTp could cause pregnancy complications.
Amoako *et al.* (2021)	48	Late ANC initiation and factors associated with sub- optimal uptake of sulpha- doxine-pyrimethamine in pregnancy: a preliminary study in Cape Coast Metropolis, Ghana.	This study sought to determine factors associated with uptake of IPTp-SP in the Metropolis that could be exploited to improve the uptake of the intervention.	Ghana	Quantitative	Pregnant women did not stop taking SP despite experiencing side effects.
Balami *et al.* (2020)	44	Determinants of uptake of first dose of intermittent preventive treatment among pregnant women in a secondary health Centre in Maiduguri, Nigeria.	This study aimed at assessing the determinants of uptake of first dose of IPTp among pregnant women at the State Specialist Hospital, Maiduguri.	Nigeria	Quantitative	Over half of those polled believed that taking all of their IPTp medications was highly beneficial to their pregnancies, while the rest had reservations to varying degrees. The positive attitudes of the women toward it could be attributed to the fact that health workers at the health facility prescribe SP, suggesting that the women had a high level of trust in the health workers, as they appeared to be confident in the safety of their medications. Only a tiny percentage of respondents said that taking IPTp was unpleasant, which is not surprising given the widespread acceptance of SP among pregnant women in Nigeria. Very few reported side effects, with the majority of them being transient and tolerable.
Boene *et al.* (2014)	36	Perceptions of malaria in pregnancy and acceptability of preventive interventions among Mozambican pregnant Women: implications for effectiveness of malaria control in pregnancy.	This study aimed at describing the perceptions and behaviours of pregnant women in relation to malaria and the acceptability of currently recommended malaria preventive interventions.	Mozambique	Mixed- methods	While some respondents thought malaria in pregnancy caused maternal death, stillbirth, hallucinations, miscarriage, congenital malaria, prematurity, and low birth weight, others did not view malaria as a threat to pregnancy. Respondents perceived SP to cause dizziness, vomiting, malaise, and nausea. Most women who experienced side effects blamed them for taking the pills too early in the morning and on an empty stomach. When delivered in a health facility, pregnant women reported high compliance with SP instructions.
Enato *et al.* (2007)	39	A survey of knowledge, attitude and practice of malaria management among pregnant women from two healthcare facilities in Nigeria.	To assess the knowledge, attitude and practice of malaria management among pregnant women attending antenatal clinics in Nigeria.	Nigeria	Quantitative	Respondents knew malaria during pregnancy could result in maternal illness, anaemia, low birth weight, abortion, and neonatal jaundice. This study also found that respondents had a low belief in the effectiveness of IPT as a malaria prevention measure during pregnancy. The majority of respondents thought anti-malarial drugs were unsafe to use during pregnancy.
Faye *et al.* (2021)	6	Participatory research for the development of information, education and communication tools to promote intermittent preventive treatment of malaria in pregnancy in the Democratic Republic of the Congo, Nigeria and Mozambique.	This study explored the attitudes, points of view, and knowledge of the participants regarding the SP packaging and the IEC tools, and the potential influence of these tools to improve the users’ perceptions of SP and their IPTp compliance.	The Democratic Republic of the Congo, Nigeria and Mozambique	Qualitative	Few women in all three countries had taken the required three SP doses during pregnancy. Contributing factors identified included women's negative perceptions of SP and side effects, which did not motivate them to take all three doses. Some women also expressed concerns about the hygiene and safety of SP. White tablets with no distinguishing features make them indistinguishable from counterfeit drugs suspected of causing pregnancy complications.
Hill *et al.* (2015)	35	Access and Use of interventions to prevent and treat malaria among pregnant women in Kenya and Mali: A qualitative Study.	Systematic examination of the operational, socio-economic and cultural constraints to pregnant women’s access to intermittent preventive treatment (IPTp), long-lasting insecticide-treated nets (LLINs) and case management in Kenya and Mali to provide empirical evidence for strategies to improve coverage.	Kenya and Mali	Qualitative	Malaria in pregnancy was widely regarded as a severe threat to pregnant women and their unborn children, causing headaches, vomiting, fever, dizziness, low birth weight, abortion, and death. Women's perspectives on the efficacy of SP were mixed. Some women claimed that SP was both effective and ineffective for malaria prevention. According to the study, the newer drugs were widely perceived to be more effective than the older ones. Although SP is no longer effective in these countries for treating malaria, it is still an effective method of malaria prevention through IPTp. Several Kenyan women believed SP was too powerful to use during pregnancy and could harm the baby or cause miscarriage. Both countries' women reported side effects from SP use, including nausea. Women in Mali reported varying degrees of side effects (malaise and dizziness) or no side effects, and they linked the vomiting associated with SP with taking the drug when hungry.
Mbonye *et al.* (2007)	41	Intermittent preventive treatment of malaria in pregnancy: Evaluation of a new delivery approach and the policy implications for malaria control in Uganda.	To assess whether CRHWs, TBAs, DSVs and APMs could provide IPT to pregnant women in rural areas, who do not easily access ANC at health units.	Uganda	Mixed- methods	The essential factor that compelled women to get the second dose of SP was the experience of improved health with the first dose. Some women reported that SP made them feel stronger and healthier and helped them have fewer malaria episodes. Women also attributed SP use to the delivery of healthy babies. Some pregnant women believe SP is a powerful drug that weakens men and has adverse effects on the foetus, such as abortions and abnormal babies. Most women reported no side effects from the first dose of SP, which encouraged them to continue with the second dose. However, some people experienced side effects from SP after taking the first dose, discouraging them from taking the second one. These women were unsure whether their signs and symptoms were caused solely by SP or other illnesses. The most frequently mentioned side effects were dizziness, nausea, vomiting, weakness, fever, minor skin rash, and stomach pain.
Mubyazi *et al.* (2005)	2	Intermittent preventive treatment of malaria during pregnancy: a qualitative study of knowledge, attitudes and practices of district health managers, antenatal care staff and pregnant women in Korogwe District, North- Eastern Tanzania.	To assess the knowledge, attitudes and practices of these groups in relation to malaria control with emphasis on IPTp services.	Tanzania	Qualitative	Some women claimed that taking SP during pregnancy could lead to abortion and skin burning, while others chose to take a lower dose than recommended.
Nyaaba *et al.* (2021)	4	A socio-ecological approach to understanding the factors influencing the uptake of intermittent preventive treatment of malaria in pregnancy (IPTp) in South- Western Nigeria.	To explore the factors contributing to the poor coverage of ITN and IPTp within a pluralistic health sector in Ogun State, southwest Nigeria.	Nigeria	Qualitative	Malaria was common during pregnancy, with adverse outcomes including miscarriages, premature births, maternal weakness, impaired foetal growth and development, and neonatal convulsions and deaths. A few pregnant women attending TBA clinics believed that pregnant women do not get malaria. Some pregnant women reported that SP use caused unwanted side effects, which they blamed for their low IPTp uptake. Swallowing difficulties, nausea, sweating, drowsiness, and frequent urination were all common complaints.
Odongo *et al.* (2014)	37	Intermittent use of sulphadoxine- pyrimethamine for malaria prevention: a cross-sectional study of knowledge and practices among Ugandan women attending an urban antenatal clinic.	To identify pregnant women’s knowledge and practices gaps as well as determine predictors of compliance with SP-IPTp, given under limited supervision.	Uganda	Quantitative	Participants recognized malaria as a leading communicable disease capable of causing stillbirths, maternal and neonatal anaemia, maternal death, fever, joint pains, fatigue, loss of appetite, and general body malaise. Some participants claimed that SP was an effective anti-malarial and that they would retake it. Others find it less effective, particularly in controlling fever, and prefer to use other medications. Some pregnant women believed SP was safe because they did not experience adverse side effects after ingesting it. However, some respondents reported unfavourable results from SP use. The most common side effects were nausea, vomiting, and general body weakness. When delivered as IPTp, pregnant women reported high compliance with SP instructions.
Onoka *et al.* (2012)	43	Low coverage of intermittent preventive treatment for malaria in pregnancy in Nigeria: demand-side influences.	To examine the influence of demand side factors on IPTp coverage.	Nigeria	Mixed- methods	SP was thought to provide malaria protection for the mother and unborn child and malaria treatment for pregnant women. Concerning the use of SP during pregnancy, while some were hesitant to take it due to potential side effects, the majority saw no reason to be concerned. They claimed that any malaria drug could cause side effects. This study discovered that women accept medicines given to them by health professionals and have no serious concerns about potential side effects.
Pell *et al.* (2014)	34	The acceptability of intermittent screening and treatment versus intermittent preventive treatment during pregnancy: results from a qualitative study in Northern Ghana.	“To explore the acceptability of IST with artemether- lumefantrine (AL) compared to IPTp with sulphadoxine- pyrimethamine (SP) and in Upper East Region, northern Ghana”.	Ghana	Qualitative	Although the participants saw SP as beneficial to themselves and their unborn children, their negative experiences with SP use frequently clouded their perception of SP benefit. Participants acknowledged the effectiveness of IPTp-SP in reducing miscarriage and improving birth weight. Some participants reported side effects from taking SP, though the side effects varied depending on the IPTp-SP dose. Women did not always experience side effects with each dose. The most commonly reported side effects were weakness, vomiting, dizziness, and diarrhoea. Despite the widespread complaints, reports of women failing to take their SP tablets were uncommon, reflecting women's general desire to follow the instructions of medical personnel despite their discomfort.
Pell *et al.* (2013)	40	Prevention and management of malaria during pregnancy: findings from a comparative qualitative study in Ghana, Kenya and Malawi.	To explore the social and cultural context to the uptake of these interventions at four sites across Africa.	Ghana, Kenya and Malawi	Qualitative	Whether pregnant women were familiar with IPTp, complaints of side effects associated with ANC medication, primarily nausea, vomiting, and dizziness, were uncommon in Kenya and central Ghana. Direct complaints about IPTp side effects, particularly vomiting, were more common in Malawi and Northern Ghana. This was despite women's greater efforts at these sites to reduce the possibility of side effects, such as nausea or vomiting, by eating a meal before receiving IPTp. However, because IPTp was widely accepted, these complaints did not always result in non-compliance.
Rassi *et al.* (2016)	38	Assessing demand‑side barriers to uptake of intermittent preventive treatment for malaria in pregnancy: a qualitative study in two regions of Uganda.	This study explored thoughts and opinions of key stakeholders (district health officials, health workers, pregnant women and mothers, opinion Leaders) to better understand how stakeholders’ perceptions may affect uptake of IPTp, particularly for those women who attend ANC.	Uganda	Qualitative	Many women stated that they were generally hesitant to take medication while pregnant but trusted nurses' and doctors' judgement and expertise. They were willing to accept medication when recommended by a health worker. Taking SP on an empty stomach appeared to be a particular concern. Respondents of all types widely reported this. Most women interviewed were aware that malaria in pregnancy could result in miscarriage and that pregnant women were more susceptible to malaria than non-pregnant women. Women agreed that pregnant women must take precautions to avoid malaria infection. Many women believed in the efficacy of SP in preventing malaria and its adverse effects on pregnant women and their unborn babies. While most of the women interviewed reported no unpleasant side effects, a few said they felt dizzy or nauseous after taking the medication. Some also reported hearing complaints about side effects from friends and family.
Tobin-West *et al.* (2013)	33	Utilization of intermittent preventive treatment of malaria by pregnant women in rivers state, Nigeria.	To assess the level of intermittent preventive treatment of malaria in pregnancy (IPTp) and to identify obstacles prohibiting its widespread use in Rivers state, Nigeria.	Nigeria	Quantitative	Most women in this study were aware that mosquito bites predispose them to malaria and that malaria could harm the mother or foetus during pregnancy, causing abortion, stillbirth, or low birth weight. Many women who attended ANC collected SP from health workers and took it home but did not use it. They claimed they did not need it because they were healthy. Some questioned the drugs' efficacy in preventing malaria during pregnancy, and others were concerned that the medicines would cause abortion or harm their unborn babies.
Tutu *et al.* (2011)	42	The effectiveness and perception of the use of sulphadoxine- pyrimethamine in intermittent preventive treatment of malaria in pregnancy programme in Offinso district of Ashanti region, Ghana.	To assess the effectiveness of SP and perception of its use in pregnant women in Offinso district (Ashanti Region), Ghana	Ghana	Mixed- methods	Most pregnant women were aware of SP and its benefits for mothers and unborn children. They claimed that SP reduces maternal morbidity, anaemia, and stillbirths and improves baby weights. Most respondents admitted that the number of pregnant women who reported being sick had decreased. The side effects experienced by the women differed depending on the IPTp-SP dose. While some pregnant women did not experience any significant side effects from SP use, a little more than a third of the pregnant women did experience minor side effects such as nausea, general malaise, body weakness, and vomiting. These effects, however, had no significant relationship with the number of SP doses taken.

## Appendix C

Below is the completed Preferred Reporting Items for Systematic reviews and Meta-Analyses extension for Scoping Reviews (PRISMA-ScR) checklist, retrieved from Tricco *et al.* [30].

**Table:** PRISMA-ScR Checklist

Section	Item	Prisma-scr * checklist item	Reported on page #
**Title**	1	Identify the report as a scoping review.	1
**Abstract**			
Structured summary	2	Provide a structured summary that includes (as applicable) background, objectives, eligibility criteria, sources of evidence, charting methods, results, and conclusions that relate to the review questions and objectives.	1
**Introduction**			
Rationale	3	Describe the rationale for the review in the context of what is already known. Explain why the review questions/objectives lend themselves to a scoping review approach.	1-2
Objectives	4	Provide an explicit statement of the questions and objectives being ad- dressed with reference to their key elements (e.g., population or participants, concepts, and context) or other relevant key elements used to conceptualize the review questions and/or objectives.	2
**Methods**			
Protocol and regis- tration	5	Indicate whether a review protocol exists; state if and where it can be ac- cessed (e.g., a Web address); and if available, provide registration infor- mation, including the registration number.	Available on request
Eligibility criteria	6	Specify characteristics of the sources of evidence used as eligibility criteria (e.g., years considered, language, and publication status), and provide a rationale.	3
Information sources*	7	Describe all information sources in the search (e.g., databases with dates of coverage and contact with authors to identify additional sources), as well as the date the most recent search was executed.	2
Search	8	Present the full electronic search strategy for at least 1 database, including any limits used, such that it could be repeated.	Appendix A
Selection of sources of evidence	9	State the process for selecting sources of evidence (i.e., screening and eli- gibility) included in the scoping review.	3
Data charting process	10	Describe the methods of charting data from the included sources of evi- dence (e.g., calibrated forms or forms that have been tested by the team before their use, and whether data charting was done independently or in duplicate) and any processes for obtaining and confirming data from investigators.	3
Data items	11	List and define all variables for which data were sought and any assump- tions and simplifications made.	3
Critical appraisal of individual sources of evidence	12	If done, provide a rationale for conducting a critical appraisal of included sources of evidence; describe the methods used and how this information was used in any data synthesis (if appropriate).	N/A
Summary measures	13	Not applicable for scoping reviews.	N/A
Synthesis of results	14	Describe the methods of handling and summarizing the data that were charted.	3
Risk of bias across studies	15	Not applicable for scoping reviews.	N/A
Additional analyses	16	Not applicable for scoping reviews.	N/A
**Results**			
Selection of sources of evidence	17	Give numbers of sources of evidence screened, assessed for eligibility, and included in the review, with reasons for exclusions at each stage, ideally using a flow diagram.	3
Characteristics of sources of evidence	18	For each source of evidence, present characteristics for which data were charted and provide the citations.	Appendix B
Critical appraisal within sources of evidence	19	If done, present data on critical appraisal of included sources of evidence (see item 12).	N/A
Results of individual sources of evidence	20	For each included source of evidence, present the relevant data that were charted that relate to the review questions and objectives.	Appendix B
Synthesis of results	21	Summarize and/or present the charting results as they relate to the re-view questions and objectives.	3-5
Risk of bias across studies	22	Not applicable for scoping reviews.	N/A
Additional analyses	23	Not applicable for scoping reviews.	N/A
**Discussion**			
Summary of evidence	24	Summarize the main results (including an overview of concepts, themes, and types of evidence available), link to the review questions and objectives, and consider the relevance to key groups.	5-6
Limitations	25	Discuss the limitations of the scoping review process.	7
Conclusions	26	Provide a general interpretation of the results with respect to the review questions and objectives, as well as potential implications and/or next steps.	7
**Funding**	27	Describe sources of funding for the included sources of evidence, as well as sources of funding for the scoping review. Describe the role of the funders of the scoping review.	N/A
*From: Tricco AC, Lillie E, explanation. Ann. Intern. Zarin Med. W, O'Brien KK, et al. PRISMA extension for scoping reviews (PRISMA-ScR): 2018, **169**:467-473. Checklist and*			
